# The Controlled Release of Abscisic Acid (ABA) Utilizing Alginate–Chitosan Gel Blends: A Synergistic Approach for an Enhanced Small-Molecule Delivery Controller

**DOI:** 10.3390/gels10030185

**Published:** 2024-03-08

**Authors:** Oscar Valdes, Daniel Bustos, Luis Guzmán, Marcelo Muñoz-Vera, Gabriela Urra, Ricardo I. Castro, Luis Morales-Quintana

**Affiliations:** 1 Centro de Investigación de Estudios Avanzados del Maule (CIEAM), Vicerrectoría de Investigación y Postgrado, Universidad Católica del Maule, Talca 3460000, Chile; ovaldes@ucm.cl; 2 Laboratorio de Bioinformática y Química Computacional, Departamento de Medicina Traslacional, Facultad de Medicina, Universidad Católica del Maule, Talca 3480094, Chile; dbustos@ucm.cl (D.B.); gabriela.urra@alu.ucm.cl (G.U.); 3 Departamento de Bioquímica Clínica e Inmunohematología, Facultad de Ciencias de la Salud, Universidad de Talca, Avenida Lircay, s/n, Casilla 747-721, Talca 3460000, Chile; lguzman@utalca.cl; 4 Multidisciplinary Agroindustry Research Laboratory, Universidad Autónoma de Chile, Cinco Pte. N° 1670, Talca 3467987, Chile; marcelomunoz132@gmail.com; 5 Multidisciplinary Agroindustry Research Laboratory, Instituto de Ciencias Aplicadas, Facultad de Arquitectura, Construcción y Medio Ambiente, Universidad Autónoma de Chile, Cinco Pte. N° 1670, Talca 3467987, Chile; 6 Multidisciplinary Agroindustry Research Laboratory, Instituto de Ciencias Biomédicas, Facultad de Ciencias de la Salud, Universidad Autónoma de Chile, Cinco Pte. N° 1670, Talca 3467987, Chile

**Keywords:** abscisic acid (ABA), alginate–chitosan blend, calcium cross-linking, delivery controller, ionic interaction

## Abstract

The integration of abscisic acid (ABA) into a chitosan–alginate gel blend unveils crucial insights into the formation and stability of these two substances. ABA, a key phytohormone in plant growth and stress responses, is strategically targeted for controlled release within these complexes. This study investigates the design and characterization of this novel controlled-release system, showcasing the potential of alginate–chitosan gel blends in ABA delivery. Computational methods, including molecular dynamics simulations, are employed to analyze the structural effects of microencapsulation, offering valuable insights into complex behavior under varying conditions. This paper focuses on the controlled release of ABA from these complexes, highlighting its strategic importance in drug delivery systems and beyond. This controlled release enables targeted and regulated ABA delivery, with far-reaching implications for pharmaceuticals, agriculture, and plant stress response studies. While acknowledging context dependency, the paper suggests that the liberation or controlled release of ABA holds promise in applications, urging further research and experimentation to validate its utility across diverse fields. Overall, this work significantly contributes to understanding the characteristics and potential applications of chitosan–alginate complexes, marking a noteworthy advancement in the field of controlled-release systems.

## 1. Introduction

Small-molecule delivery systems have revolutionized materials science, agriculture, and biomedical research, offering an unparalleled ability to finely control the release kinetics of bioactive compounds [1,2,3]. Biopolymer-based controlled-release systems, among various strategies, have gained significant attention [4,5,6,7].

These systems hold promise in diverse applications, from drug delivery to agriculture, providing opportunities to optimize therapeutic outcomes and agricultural practices [3,8]. One such bioactive molecule is abscisic acid (ABA), a phytohormone crucial in plant growth, development, and stress responses [9], especially in nonclimacteric fruits like strawberries [10,11,12]. The precise control of bioactive compound release, including hormones like ABA acting as signaling molecules, holds immense potential for the future of agriculture and other industries [8]. Controlled-release systems play a pivotal role in optimizing the efficacy and therapeutic outcomes of these compounds [13]. The intentional design of such systems not only advances our understanding of molecular interactions in complex matrices but also holds promise in applications in agriculture, pharmaceuticals, and beyond [14].

In this context, our study explores the design and characterization of a novel controlled-release system tailored for ABA [1]. Two biopolymers, alginate and chitosan, have emerged as promising candidates. These natural fibers are renowned for their biodegradability, biocompatibility, non-toxicity, and cost-effectiveness [3,15,16].

Alginate, which consists of alternating L-guluronate (G) and D-mannuronate (M) units, creates a polymeric mesh or gel by engaging in ionic exchange with calcium ions [8,17]. Chitosan, characterized as a weak cationic polysaccharide predominantly composed of (1,4)-linked 2-amino-2-deoxy-D-glucan units, has the capability to form ionic bonds with structures that possess carboxylic acids, such as alginates [18]. The combination of chitosan and alginate stands out as a well-established and efficacious technique to enhance the performance of polymer materials [1,10]. This gel blending facilitates the formation of systems through interpolyelectrolyte complex reactions, resulting in properties like release-retarding behavior, primarily induced through alginate–chitosan complexation and calcium cross-linking.

The gelation process begins by introducing a solution of calcium chloride to a solution of alginate and chitosan. Calcium ions interact with the carboxylic acid groups of alginates and the amine groups of chitosan, forming a stable gel network [19]. Using calcium ions as cross-linkers for both alginate and chitosan has proven effective for the controlled release of bioactive compounds [20]. Recognizing the importance of precisely regulating the release of bioactive compounds, including hormones and signaling molecules, our study delves into the design and characterization of a novel controlled-release system specifically tailored for ABA; it can serve as a starting point to apply this phytohormone to soils in the future and determine its effect on plants.

As we explore the intricate details of the design, molecular dynamics simulations, and experimental validations, this study aims to highlight the potential of alginate–chitosan matrices as effective carriers for the controlled release of ABA, unlocking new possibilities for precision in small-molecule delivery. This article provides an in-depth analysis of controlled-release systems based on an alginate and chitosan gel blend, employing a synergistic combination of these well-established biopolymers known for their properties [1].

## 2. Results and Discussion

### 2.1. Morphological Analysis by Scanning Electron Microscopy (SEM)

**Figure 1:**
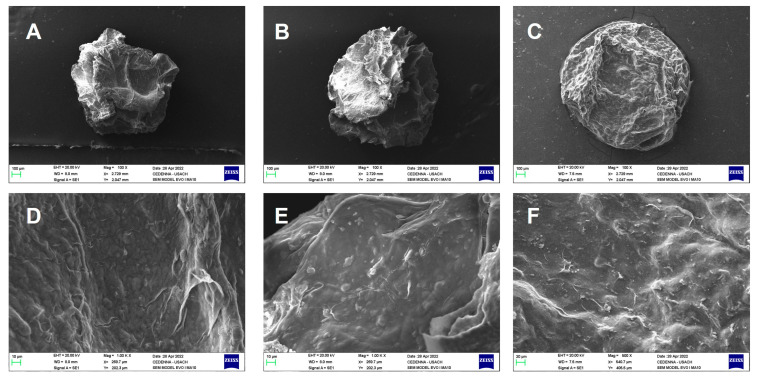
SEM images of alginate–chitosan–ABA Ca^2+^ blend: 100× magnification images of the cross-linked alginate–chitosan–abscisic acid blend gel for delivery system, with alginate/chitosan ratios of (**A**) 2:1, (**B**) 1:1, and (**C**) 1:2. Ratios of alginate/chitosan in the 500× magnification images are (**D**) 2:1, (**E**) 1:1, (**F**) 1:2.

Figure 1 displays scanning electron microscopy (SEM) images of the dual cross-linked beads with various mass ratios that were obtained. These images reveal the surface and internal morphology of the ALG-CS (alginate–chitosan–ABA) formations with mass ratios of 1:2, 1:1, and 2:1. Notably, as the mass ratio of chitosan decreases, the bead surfaces become smoother. In the case of the SEM image in Figure 1A and its enlargement in Figure 1F, a significant presence of wrinkles on the surface can be observed, a result of the interaction between ALG and CS. These results were described by Lai et al. (2003) [21], who proposed an explanation for this phenomenon, indicating that the blend system could give rise to a random fibrillar network [21]. Additionally, with an increase in the mass ratio of alginate, the degree of compaction in the beads intensifies (see Figure 1B,C) [22]. This suggests that the concentration of alginate in the blend significantly influences the compactness of the bead structure, affecting its porosity and, consequently, its potential applications in controlled-release systems and other relevant fields.

### 2.2. Thermogravimetric Analysis

An improvement in thermal stability is highlighted with the formation of gel blends between alginate and chitosan, especially when using a cross-linking agent, in comparison to the stability of alginate alone. However, the blends of both polymers prevent this cross-linking from occurring. This is due to the interaction of alginate acids with chitosan amines, generating ionic bonds between groups of -CH2COO^−^ in binding sites, interacting with chitosan through its -NH_3_^+^ groups, reducing the stability of the blends compared to chitosan alone [23].

This hinders the formation of more compact and ordered structures, such as those formed by alginate with bivalent ions, resulting in well-organized structures known as egg-box structures [24,25].

**Figure 2:**
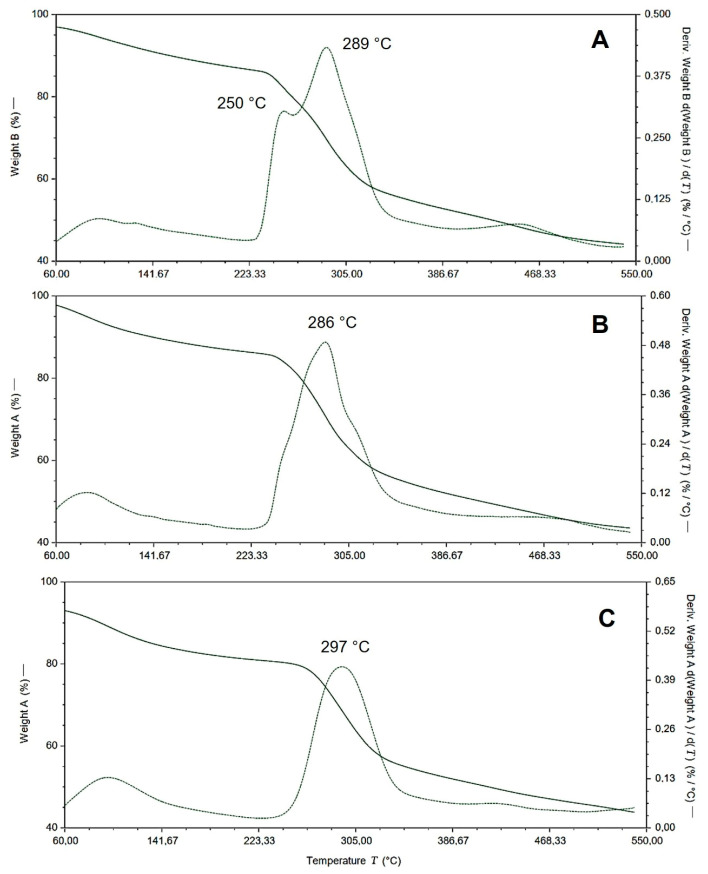
Thermogravimetric analysis (TGA) and the differential thermogravimetric analysis (DTG) thermogram for the polymer complexes: chitosan and alginate gel blends at different ratios (alginate/chitosan) of (**A**) 2:1, (**B**) 1:1, (**C**) 1:2.

The blends’ structures and their different thermal stabilities are clearly depicted in Figure 2. The ALG/CS 2:1 gel blend degraded around 250 °C due to alginate chains, associated with the dehydration of saccharide rings, disruption of C–H bonds, and breakage of glycosidic C–O–C bonds in the main chain of alginate [26]. However, Figure 2 also shows a stability peak at 289 °C. This improvement is attributed to the formation of cross-links facilitated by cross-linking agents like CaCl_2_, contributing to higher thermal stability. In contrast, the ALG/CS ratios of 1:2 and 1:1 exhibited better stability compared to the gel blend with the ALG/CS 2:1 structure due to the presence of more chitosan chains. This is attributed to the formation of cross-linkers between calcium ions with alginate and the formation of ionic bonds between the acids of alginate and the chitosan amines.

### 2.3. Characterization of Complex by ATR-FTIR Spectroscopy

**Figure 3:**
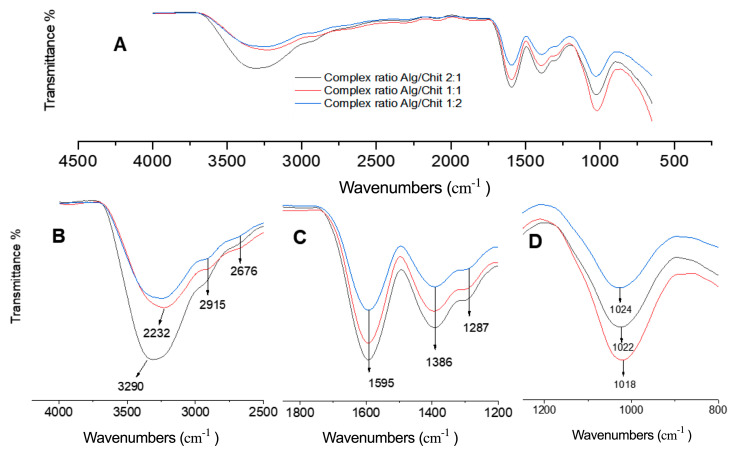
The vibrational frequencies (cm^−1^) obtained using FT-IR of the different complexes. (**A**) Spectra of different blends; (**B**) spectra range 4000 at 2600 cm^−1^; (**C**) spectra range 1800 at 1200 cm^−1^; and (**D**) spectra range 1200 at 800 cm^−1^.

Using ATR-FTIR spectroscopy, we characterized the complex. By comparing the spectra, it was evident that ABA, alginate, chitosan, and calcium could form stable complexes, effectively incorporating ABA molecules within their interstitial spaces [14]. In Figure 3A, the FTIR spectrum illustrates the functional groups of the complex. Notably, stretching of the hydroxyl group (OH) in chitosan and alginate is observed at 3290 and 3232 cm^−1^, respectively (see Figure 3B) [27,28].

Moreover, the FTIR spectrum typically reveals peaks associated with the stretching vibration of the amino group (NH_2_) at 3290 cm^−1^ (Figure 3A) [29] and the C-O-C stretching vibration at 1018, 1020, and 1022 cm^−1^ [Figure 3D]. An overlap occurs between the N-H bending of the amino group in chitosan and the carboxylate ion vibration of alginate [30] (see Figure 3C).

Additionally, the FTIR spectrum of alginate and chitosan displays peaks corresponding to the antisymmetric stretching vibration of the C-O-C bond near 1020 cm^−1^ and the stretching vibration of the carboxylate ion COO- at 1578 cm^−1^, indicating the presence of alginate.

Lastly, the distinct peaks at 2915 cm^−1^ and 2676 cm^−1^ signify the presence of asymmetric CH stretching and symmetric CH stretching, respectively, indicating the successful incorporation of ABA molecules within the complexes. This comprehensive spectral analysis enhances our understanding of the complex structure and its constituent functional groups.

### 2.4. In Vitro Release Studies

**Figure 4:**
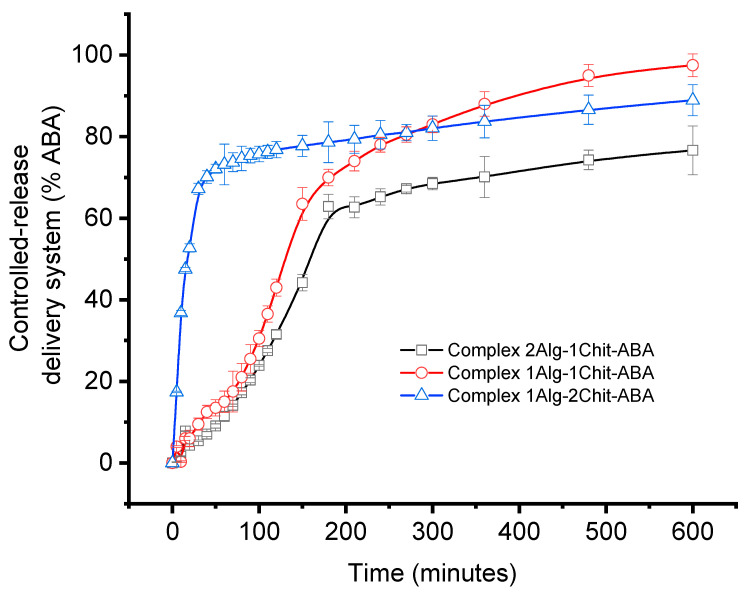
Cumulative release of model compounds from alginate–chitosan–ABA complex using different formulations in phosphate buffer solution at pH 7.4; alginate/chitosan ratios of (2:1 (black curve, system 1), 1:1 (red curve, system 2), 1:2 (blue curve, system 3). Data are presented as average ± SEM (n = 3).

Drug release from spherical polymeric materials was affected by the swelling degree, pH, ionic strength, and temperature. Figure 4 shows cumulative ABA release percentage curves of the three types of microparticles in a phosphate buffer solution (pH 7.4 at 25 ± 0.5 °C as a function of time), and Table 1 shows the total ABA in the different complexes. As can be observed, all composites show the characteristic release profile for these kinds of materials. The highest cumulative release rate was obtained for system 3, corresponding to the microparticles with the maximum amount of chitosan. In contrast, system 1, with the lowest percentage of chitosan and alginate, had the slowest ABA release rate. This behavior could be due to the preparation method of the microparticles, which, in this study, was a one-step method. In the one-step method, the alginate network is formed by the reaction with both chitosan and calcium ions, allowing the diffusion of chitosan molecules into the alginate gel core. The concentration of the chitosan solution during the particle preparation greatly affected the ability of calcium ions to find the guluronic chains in the alginate, directly affecting cross-linking. Their results suggested a higher diffusion rate of chitosan molecules into the alginate core in the presence of calcium chloride concentrations, resulting in a higher porosity of the alginate core, favoring the release profile of ABA [22,31]. The opposite occurred with system 1, where the lower amount of chitosan improved the alginate cross-linking, producing a denser network in the microparticles, limiting the ABA diffusion. In conclusion, the drug release rate from the film formulations can be ordered as follows: system 2 > system 3 > system 1.

### 2.5. Kinetic Swelling Study

Mathematical models have always been one of the most effective ways to determine drug release mechanisms and release kinetics for various systems [32]. For this purpose, we will discuss using four different mathematical models for fitting the experimental data. The model parameters were calculated using the nonlinear least squares regression method. The *RSS* values indicate a better fit of experimental data into the model. It is essential to mention that all model parameters were calculated when the release of the drug reached 60 percent of the total content [33].

**Figure 5:**
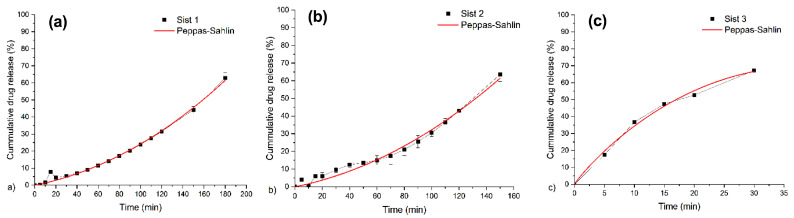
Fitting of appropriate experimental data (M_t_/M_∞_ ≤ 0.6) to Peppas–Sahlin kinetic model for system 1 (**a**), system 2 (**b**), and system 3 (**c**).

Table 2 shows parameters such as the release kinetic constant (k), diffusional exponent (n), kinetic constants of the diffusion (k_D_) and relaxation (k_R_), and the burst effect associated with drug delivery from the matrix surfaces (b), corresponding to the four release models, respectively. According to the values obtained for the residual sum of the squares (*RSS* > 0.99) reported in Table 2, Peppas–Sahlin is the best-fit model for the experimental data for all systems [34]. It is known that Peppas–Sahlin is an advantageous model to explain when more than one mechanism is involved in the drug release or when accurate mechanisms are unknown (Figure 5). The values of n reported in Table 2 are around 1 for all samples, indicating that the ABA release happens via Super Case II transport. In the cases of systems 1 and 2, the chain relaxation process has more influence than the diffusion process, which is reflected in the k_R_ values (positive and increasing with decreasing sodium alginate content) and k_D_ (around 0 and without a trend). On the contrary, for system 3, the diffusion process has more influence than the chain relaxations, which is reflected in the k_D_ values due to the chitosan content (Figure 5).

### 2.6. In Silico ABA Delivery Evaluations

**Figure 6:**
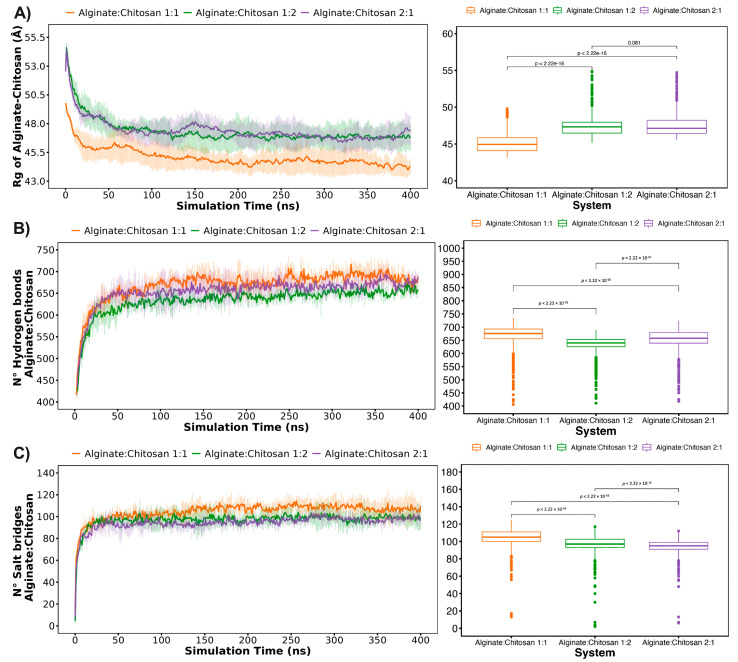
Stabilization of the nanoparticles over the simulation time: (**A**) radius of gyration of alginate and chitosan, (**B**) the number of hydrogen bonds between alginate and chitosan, and (**C**) the number of salt bridges between the ALG/CS complex and ABA.

**Figure 7:**
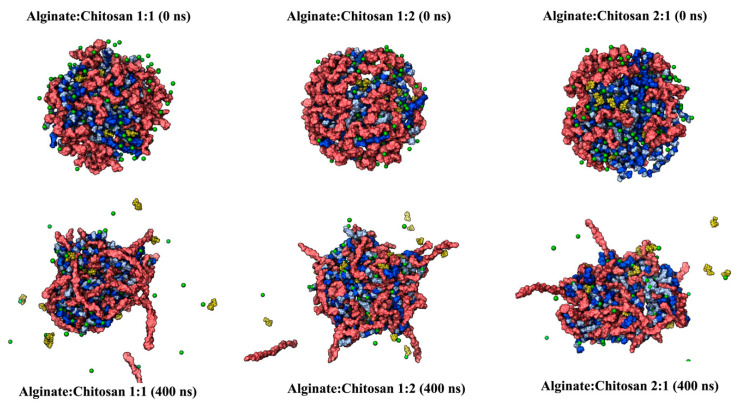
Alginate–chitosan–ABA nanoparticle formation. The initial (0 ns) and final (400 ns) time steps for each complex are depicted. Both types of alginate chains are colored in blue and cyan (in the core of the molecules), while chitosan chains and ABA molecules are in red and yellow colors, respectively.

Molecular dynamics (MD) simulation studies were conducted to investigate the interaction and the controlled delivery mode between the ALG/CS complexes and ABA in an aqueous environment. Firstly, the radius of gyration (Figure 6A) and the intermolecular interactions between alginate and chitosan chains (Figure 6B,C) measured by hydron bonds and salt bridges agree, showing that the 1:1 system (alginate–chitosan) is more compact and stable due to the parity in the number of chains of both polymers of opposite electrostatic nature. On the other hand, the 1:2 and 2:1 configurations tended to be less compact due to having a different number of alginate and chitosan chains after 400 ns of the simulations (Figure 7).

**Figure 8:**
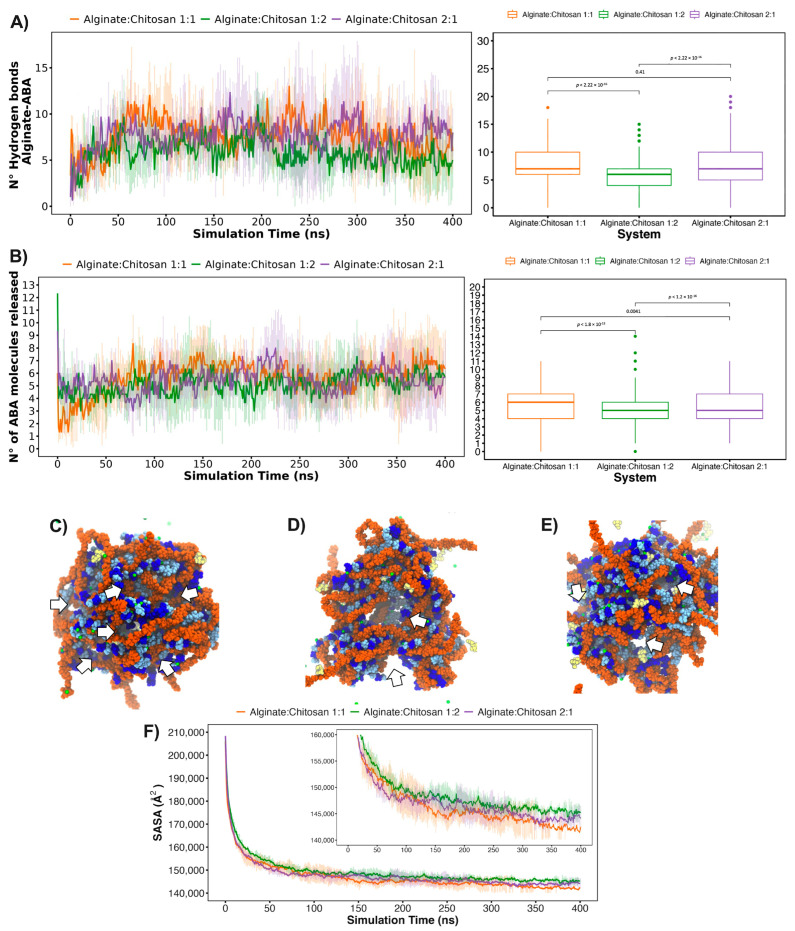
Molecular characterization of nanoparticles and ABA. The graph depicts (**A**) intermolecular hydrogen bond interactions between the alginate nanoparticles and ABA over the simulation period, and (**B**) the release profile of ABA molecules from the polymer. Statistical analyses for each molecular descriptor are included. In (**C**–**E**), the final states of nanoparticles are presented for the 1:1, 1:2, and 2:1 alginate/chitosan ratios, respectively, with white arrows indicating porosities formed by polymer composition ratios during simulation. Dark blue and light blue represent alginate monomers β-D-mannopyranuronate and α-L-gulopyranuronate, orange denotes chitosan chains, yellow signifies ABA, and green represents calcium ions. (**F**) The solvent accessible surface area (SASA) is illustrated across the simulation for the three studied proportions. The inset offers an approach to discerning SASA differences between the systems from ~25 ns onward.

Additionally, the quantity of hydrogen bonds (HBs) formed between the ALG/CS systems and ABA (Figure 8A) is closely linked to the experimental findings concerning cumulative drug release. This connection arises from the expectation that the rate of drug release is inversely proportional to the number of interactions established between the nanoparticle and the drug. Specifically, the 1:2 alginate–chitosan system, having the fewest interactions, is anticipated to exhibit the fastest drug release. Following this, the 1:1 alginate–chitosan system, which boasts an intermediate number of interactions, is expected to release the drug at a moderate pace. Lastly, the 2:1 system, characterized by the highest number of nanoparticle–ABA hydrogen bond interactions, is projected to release the drug at a slower rate. The ABA release profiles over the 400 ns simulation period, as depicted in Figure 7, demonstrate generally similar behavior across all three systems, even though the number of alginate and chitosan nanoparticles is different in each system. In this context, it is noteworthy that the 1:2 system exhibits a notable presence of outliers, with some instances reaching a maximum of 15 molecules released, equivalent to 75% of total release (Figure 8C–E). In contrast, the other systems achieve approximately 50% release under similar conditions (Figure 8C–E). The SASA (solvent-accessible surface area) was employed as a metric to assess the porosity of the examined systems. Figure 8F illustrates that the 1:2 system exhibits a higher degree of accessibility to the solvent. Furthermore, the final frames of all three systems are presented, revealing that the 1:2 system displays significantly larger porosities, denoted by the white arrows, compared to the other two systems. This observation offers Insight into the factors contributing to the higher release rate in the 1:2 system.

In comparison to the computational methods employed in this study, a comprehensive analysis of molecular dynamics simulations and computational techniques was presented in a related previous study by the same group and described by Bustos et al. (2023) [1]. The computational framework was outlined in the work of Bustos et al. (2023) [1]. Notably, the simulation methods in both studies share a fundamental reliance on sophisticated software tools for molecular dynamics simulations. The incorporation of these insights refines our understanding of the computational approaches, offering complementary perspectives on the molecular interactions within the studied systems. While the present study contributes valuable findings, the integration of methodologies from the present study enriches our comprehension of the intricacies involved in our computational simulations, providing a more holistic view of the molecular dynamics and their implications in chitosan–alginate complexes.

**Figure 9:**
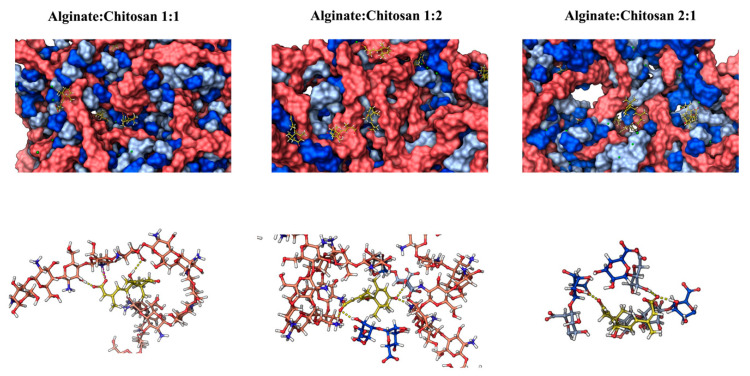
Approach to the intermolecular cavities of the ALG/CS nanoparticle where the ABA molecules are interacting with alginate/chitosan chains.

The release of ABA is described in the context of the formation, stability, and characterization of chitosan–alginate complexes (Figure 9). The release of ABA is a crucial aspect, particularly in drug delivery systems. ABA is known for its role in various physiological processes, including the stress response in plants. In the present paper, there is a focus on the computational methods used to study the interactions within the complexes. Molecular dynamics simulations, such as those performed with the Schrödinger software (MAESTRO, 2021), provide insights into the structural effects of pH variation and calcium concentration on the microencapsulation process. These computational methods contribute to a deeper understanding of the behavior of the chitosan–alginate–ABA complexes. Considering these aspects, the liberation, or controlled release, of ABA from chitosan–alginate complexes appears to be a strategically important method, especially in the context of drug delivery systems. Liberation allows for the targeted and regulated release of ABA, which can have implications in applications beyond drug delivery, such as in the study of plant stress responses. It enables the controlled delivery of ABA, providing avenues for applications in various fields, including pharmaceuticals and agriculture. However, the effectiveness of this method depends on the specific context, and further research and experimentation would be necessary to fully validate its utility in different applications.

## 3. Conclusions

The present work showed the successful integration of abscisic acid (ABA) into the interstitial spaces of chitosan–alginate complexes. This integration is crucial for the overall stability and formation of these complexes, shedding light on their characteristics. The study significantly contributes to a broader understanding of the formation and stability of chitosan–alginate complexes, emphasizing their potential applications, especially in drug delivery systems. The integration of ABA into chitosan–alginate complexes is presented with potential implications for drug delivery systems, suggesting a possible approach for controlled-release strategies and targeted delivery.

## 4. Material and Methods

### 4.1. Materials to Form the Molecular Blend

Chitosan with a molecular weight of 160 KDa (medium viscosity) was procured from Sigma-Aldrich. The abscisic acid (ABA) employed, consisting of mixed (+/−) sis-trans isomers, was obtained from Phytotechlab. Water with a purity of 18.2 MΩ·cm, known as Milli-Q water, and calcium chloride hexahydrate (98% purity) were sourced from Sigma-Aldrich. All additional chemicals utilized in the study were of reagent-grade quality.

### 4.2. Gel Blend Preparation

For the preparation of the sodium alginate and chitosan gel blend, the specific procedure was adhered to was described by Castro et al. [1]. Initially, 1.5 g of each polymer was accurately weighed and dissolved in 100 mL of Milli-Q water (1.5%, *w*/*v*). The dissolution of the solutions took place at room temperature with continuous mechanical stirring overnight, ensuring a comprehensive mixing process and complete hydration of the polymers, resulting in well-blended homogeneous solutions. Additionally, 15 mg of ABA was precisely measured and dissolved in 10 mL of Milli-Q water. After this, distinct complexes were created by combining chitosan, alginate, and abscisic acid at alginate/chitosan ratios of 2:1, 1:1, and 1:2. To introduce the bioactive component, 1 mL of abscisic acid (ABA) was incorporated into various alginate and chitosan blend solutions, resulting in the formation of complexes. The blend solutions were carefully transferred to a drop of 20 mL with an 18 G injection needle (outer diameter = 1.27 mm) and added drop by drop to a 2% (*w*/*v*) calcium chloride solution. Spherical beads were formed through mechanical stirring for 15 min, followed by a thorough wash with Milli-Q water before being air-dried at room temperature.

### 4.3. Scanning Electron Microscopy (SEM)

The samples were prepared for scanning electron microscopy by affixing them to a pin holder using carbon tape. Each sample was then coated with a 30-nanometer Au layer using a Cressington 108 Auto Sputter Coater to prevent the charging process from occurring during the observation. At 20 kV, the morphology was investigated with a Carl Zeiss EVO MA 10 scanning electron microscope.

### 4.4. Thermogravimetric Analysis (TGA)

The complex’s stability was determined using samples dried in a lyophilization apparatus (model BK-FD10P, freezer-DRYER) from a pressure chamber. The thermogravimetric analysis (TGA) of all samples was conducted using 5 mg of sample in an STD 650 thermal analyzer (TA instrument) to determine the stability. Each sample was heated at a constant rate of 10 °C min^−1^ using air as the reactive gas and a mass flow of 50 mL min^−1^. In addition, 50 mL min1 of N_2_ was used in the electronic balance as a protective gas.

### 4.5. Attenuated Total Reflection–Fourier-Transform Infrared Spectroscopy (ATR-FTIR Spectroscopy)

The samples underwent meticulous preparation and subsequent analysis through Fourier-Transform Infrared (FTIR) spectroscopy (Cary-360 instrument from Agilent Scientific Instruments, Santa Clara, CA, USA). The instrument was equipped with an Attenuated Total Reflection (ATR) module. Absorbance measurements were conducted across the range of 500 to 4000 cm^−1^, with the resolution set at 4 cm^−1^.

### 4.6. Quantification of Abscisic Acid (ABA)

For the total quantification, complex flasks containing 4 mg of sample (dry) in 5 mL of Milli-Q water were immersed in an ultrasonic bath and sonicated at a frequency of 50 kHz with a power of 100 W for 1 h to determine the amount of ABA and the mechanism of drug release. An aliquot of the supernatant was then extracted, and the content of ABA was quantified spectrophotometrically at a wavenumber of 250 nm. The quantification was accomplished by constructing a calibration curve as a function of the linear range for the determination of ABA concentrations of 20, 12, 8, and 4 mg L^−1^; for the quantification of the controlled release of ABA in the complex, the outcomes of the controlled release were determined from the overall content of ABA present in the system. The percentage of controlled release was expressed for all measurements, and to ensure accuracy, all the analyzes were conducted in triplicate.

### 4.7. Sustained Release of Abscisic Acid (ABA) and Release Kinetics Study

To study the ABA release mechanism from different types of microparticles, four different kinetic models were used to analyze the experimental data:

Mt/M∞ = kt_1/2_ (Higuchi release model). (1)

Mt/M∞ = ktn (Ritger–Peppas release model).(2)

Mt/M∞ = kDtn + b (Lindner–Lippold release model).(3)

Mt/M∞ = kDtn + kRt_2_n (Peppas–Sahlin release model). (4)

where Mt/M∞ is the fractional drug release; t is the release time; k is a release kinetic constant; n is the diffusional exponent; kD and kR are the kinetic constants of the diffusion and relaxation process; and the b term represents the burst effect associated with drug delivery from the matrix surfaces, respectively.

It is important to note that the diffusional exponent indicates the drug release mechanism. Specifically, for a spherical delivery system, when n = 0.43, the drug release mechanism is Fickian diffusion. When n = 0.85, the drug release mechanism is considered to be Case II transport, leading to zero-order release. When the value of n is between 0.43 and 0.85, anomalous transport is observed. Finally, when n > 0.85, Super Case II transport occurs [35]. All the above mathematical models are only valid for the first 60% of the drug release. Experimental data were analyzed by linear and nonlinear least squares regression using OriginPro 2021 v9.8.0.200 software (OriginLab Corp, Northampton, MA, USA). The residual sum of the squares (RSS) was calculated to distinguish the best model that described the experimental data [36].

### 4.8. Computational Assays

For molecular-level characterization, the three distinct systems with alginate/chitosan ratios of 1:1, 1:2, and 2:1 underwent energy minimization using the parameters outlined by Bustos et al. (2023) [1]. Subsequently, the Desmond/Maestro (Schrödinger) suite [37] and the OPLS v.2005 [38] were employed as the force field for simulation. The default relaxation protocol, involving five brief simulations, was applied based on the procedures specified in Bustos et al. (2023) [1]. The production simulations were executed in an NPT ensemble under standard conditions (pressure = 1 Atm, temperature = 300 °K), each lasting 400 ns, and conducted in triplicate.

## Figures and Tables

**Table 1:** Quantification of the ABA concentration in each complex.

Sample	Complex + ABA		
Alginate/Chitosan Ratio	2:1 (System 1)	1:1 (System 2)	1:2 (System 3)
Concentration of ABA in the complex per 0.9 mg of complex	0.035 ± 0.002	0.029 ± 0.003	0.012 ± 0.001

**Table 2:** Estimated parameters and *RSS* values obtained from fitting experimental data to Higuchi, Ritger–Peppas, Lindner–Lippold, and Peppas–Sahlin release models.

**Higuchi**					
	*k*				*RSS*
System 1	2.6590 ± 0.2900				0.6701
System 2	3.0688 ± 0.3061				0.7028
System 3	11.7861 ± 0.4767				0.9699
**Ritger–Peppas**					
	*n*		*k*		*RSS*
System 1	−61.8348 ± 8.9384 × 10^42^		−22.4446 ± 1.9096 × 10^41^		1.0622
System 2	1.5041 ± 0.0929		0.0321 ± 0.0144		0.9753
System 3	0.6340 ± 0.0642		7.9518 ± 1.5493		0.9864
**Lindner–Lippold**					
	*n*	*k_D_*		*b*	*RSS*
System 1	−5.6696 × 10^−4^ ± 0.3957	−23,235.3678 ± 1.6186 × 10^7^		23,202.3017 ± 1.6183 × 10^7^	0.6628
System 2	1.8102 ± 0.1195	0.0067 ± 0.0040		3.5523 ± 0.9965	0.9863
System 3	0.6228 ± 0.0886	8.3559 ± 2.6245		−0.8131 ± 3.6201	0.9867
**Peppas–Sahlin**					
	*n*	*k_D_*		*k_R_*	*RSS*
System 1	1.0690 ± 0.8637	0.1037 ± 0.1977		5.2375 × 10^−4^ ± 0.0058	0.9912
System 2	1.0730 ± 1.3880	0.1323 ± 0.4052		7.0062 × 10^−4^ ± 0.0124	0.9933
System 3	0.9205 ± 0.1342	4.8247 ± 1.5454		−0.0836 ± 0.0481	0.9936

## Data Availability

The data presented in this study are openly available in article.
